# Polyclonal human antibodies against glycans bearing red meat-derived non-human sialic acid *N*-glycolylneuraminic acid are stable, reproducible, complex and vary between individuals: Total antibody levels are associated with colorectal cancer risk

**DOI:** 10.1371/journal.pone.0197464

**Published:** 2018-06-18

**Authors:** Annie N. Samraj, Kimberly A. Bertrand, Robert Luben, Zahra Khedri, Hai Yu, Dzung Nguyen, Christopher J. Gregg, Sandra L. Diaz, Sherilyn Sawyer, Xi Chen, Heather Eliassen, Vered Padler-Karavani, Kana Wu, Kay-Tee Khaw, Walter Willett, Ajit Varki

**Affiliations:** 1 Department of Medicine, University of California, San Diego, California, United States of America; 2 Department of Cellular & Molecular Medicine, Glycobiology Research and Training Center, University of California, San Diego, California, United States of America; 3 Slone Epidemiology Center, Boston University, Boston, Massachusetts, United States of America; 4 Department of Public Health and Primary Care, University of Cambridge, Cambridge, United Kingdom; 5 Department of Chemistry, University of California, Davis, California, United States of America; 6 Channing Division of Network Medicine, Brigham and Women’s Hospital and Harvard Medical School, Boston, Massachusetts, United States of America; 7 Department of Epidemiology, Harvard T.H. Chan School of Public Health, Boston, Massachusetts, United States of America; 8 Department of Nutrition, Harvard T.H. Chan School of Public Health, Boston, Massachusetts, United States of America; University of Liverpool, UNITED KINGDOM

## Abstract

**Background:**

*N*-glycolylneuraminic acid (Neu5Gc) is a non-human red-meat-derived sialic acid immunogenic to humans. Neu5Gc can be metabolically incorporated into glycan chains on human endothelial and epithelial surfaces. This represents the first example of a “xeno-autoantigen”, against which circulating human “xeno-autoantibodies” can react. The resulting inflammation (“xenosialitis”) has been demonstrated in human-like Neu5Gc-deficient mice and contributed to carcinoma progression via antibody-mediated inflammation. Anti-Neu5Gc antibodies have potential as biomarkers for diseases associated with red meat consumption such as carcinomas, atherosclerosis, and type 2 diabetes.

**Methods:**

ELISA assays measured antibodies against Neu5Gc or Neu5Gc-glycans in plasma or serum samples from the Nurses’ Health Studies, the Health Professionals Follow-up Study, and the European Prospective Investigation into Cancer and Nutrition, including inter-assay reproducibility, stability with delayed sample processing, and within-person reproducibility over 1–3 years in archived samples. We also assessed associations between antibody levels and coronary artery disease risk (CAD) or red meat intake. A glycan microarray was used to detected antibodies against multiple Neu5Gc-glycan epitopes. A nested case-control study design assessed the association between total anti-Neu5Gc antibodies detected in the glycan array assay and the risk of colorectal cancer (CRC).

**Results:**

ELISA assays showed a wide range of anti-Neu5Gc responses and good inter-assay reproducibility, stability with delayed sample processing, and within-person reproducibility over time, but these antibody levels did not correlate with CAD risk or red meat intake. Antibodies against Neu5Gc alone or against individual Neu5Gc-bearing epitopes were also not associated with colorectal cancer (CRC) risk. However, a sialoglycan microarray study demonstrated positive association with CRC risk when the total antibody responses against all Neu5Gc-glycans were combined. Individuals in the top quartile of total anti-Neu5Gc IgG antibody concentrations had nearly three times the risk compared to those in the bottom quartile (Multivariate Odds Ratio comparing top to bottom quartile: 2.98, 95% CI: 0.80, 11.1; P for trend = 0.02).

**Conclusions:**

Further work harnessing the utility of these anti-Neu5Gc antibodies as biomarkers in red meat-associated diseases must consider diversity in individual antibody profiles against different Neu5Gc-bearing glycans. Traditional ELISA assays for antibodies directed against Neu5Gc alone, or against specific Neu5Gc-glycans may not be adequate to define risk associations. Our finding of a positive association of total anti-Neu5Gc antibodies with CRC risk also warrants confirmation in larger prospective studies.

## Introduction

Altered cell surface glycosylation is common feature in human cancers [1,2]. Expression of such tumor-associated glycan antigens results in altered cell phenotypes, and can sometimes elicit an antibody response [3–6]. One such alteration involves expression of *N*-glycolylneuraminic acid (Neu5Gc), a common mammalian cell surface sialic acid once thought to be an oncofetal antigen in humans [7] that was associated with “Hanganitzu-Deicher” antibodies (which agglutinated animal red cells) and occurred only in cancer and certain other diseases [8]. However, this “H-D” antigen is now known to be defined by the non-human sialic acid Neu5Gc, which enters into the human body and is displayed on endothelial and epithelial cell surfaces via metabolic incorporation from dietary sources, which are principally “red meats”, such as beef, pork and lamb [9].

Sialic acids including Neu5Gc are typically located at the outer termini of cell surface glycan chains in vertebrates [10,11]. These nine-carbon monosaccharides have a remarkable potential for diversity in structure, glycosidic linkage, underlying glycans, and a multitude of natural modifications [10,11]. The C-5 position in *N*-acetylneuraminic acid (Neu5Ac, the most common sialic acid form), has an *N*-acetyl group. This 5-*N*-acetyl group on cytidine 5'-monophosphate-Neu5Ac (CMP-Neu5Ac, the activated donor for sialyltransferases) can be hydroxylated by cytidine 5’-monophosphate-Neu5Ac hydroxylase (CMAH), to produce CMP-Neu5Gc [12–14]. While Neu5Ac and Neu5Gc are the two major sialic acid forms in most mammals, Neu5Gc cannot be synthesized by humans [15,16], due to an Alu-Alu fusion-mediated deletion of exon 6 in the *CMAH* gene, which resulted in highly truncated and inactive CMAH enzyme [17]. However, human cells are capable of metabolically incorporating exogenously provided Neu5Gc and presenting it on cell surface glycans as if it was synthesized in the same cell [18]. In keeping with this unusual “Trojan horse” mechanism, consumption of Neu5Gc-rich foods (mainly red meats), leads to metabolic incorporation of Neu5Gc on cell surfaces of human tissues such as epithelia and endothelia [9], and more prominently into epithelial cancers (carcinomas) [8] and atheromatous lesions [19].

Given that Neu5Gc is a “xeno-autoantigen”, it is not surprising that most humans tested to date have circulating anti-Neu5Gc polyclonal antibodies (“xeno-autoantibodies”), the majority of which are typically of the IgG isotype, and directed against a spectrum of Neu5Gc-containing glycans [20]. These antibodies first appear in infants at ~6 months of age, coinciding with Neu5Gc introduction in the diet, and reach the adult levels by 1 year of age [21]. However, these antibodies are likely not induced by dietary gut exposure, but rather via Neu5Gc scavenging by commensal bacteria, such as *Haemophilus influenzae*, that can metabolically incorporate and express the immunogenic Neu5Gc into their cell wall lipooligosaccharides, and thus appear to immunize the host to generate anti-Neu5Gc IgM and IgG antibodies [21]. Anti-Neu5Gc antibody levels in some individuals can be as high as ~0.1–0.2% of total IgG immunoglobulins, approaching levels of other major anti-glycan antibodies in normal human sera, e.g. anti-ABO blood group and anti-α-Gal antibodies [20].

Metabolically incorporated Neu5Gc displayed on human cell surfaces can interact with circulating anti-Neu5Gc antibodies and mediate chronic inflammation termed “xenosialitis”, potentially relevant in the progression of diseases associated with chronic inflammation including cancer, cardiovascular disease, and autoimmunity [22]. Notably, the primary dietary source of Neu5Gc is red meat (muscle of mammalian origin), a known risk factor also for diseases associated with chronic inflammation such as carcinomas (particularly colorectal cancer), coronary heart disease, stroke, metabolic syndrome and type 2 diabetes [23–36]. Additionally, growing evidence indicates that high red meat intake is associated with increased all-cause mortality [37–40].

There are many suggested mechanisms of the red meat-associated disease risk, including mutagens resulting from high temperature cooking [41], preservatives in processed meats [42], heme iron [40,43], trimethylamine-*N*-oxide (TMAO) stemming from gut microbiota metabolism of L-carnitine in red meat [44] and more recently recognized viruses in certain red meats [45]. However, as reviewed elsewhere [46], experimental and observational studies addressing most of these theories are inconclusive. Furthermore, other than the viral theory, and the heme theory (possibly for CRC only) most of the suggested mechanisms are not even specific to red meat, e.g., cooking derived mutagens and preservatives are also associated with poultry and fish, and the major dietary source of TMAO is not red meat. Also unexplained is the human-specificity of the risk, with other habitual carnivores being apparently unaffected. Finally, most of the proposed carcinogenic mechanisms may not be applicable to the increased risk of atherosclerosis and type 2 diabetes associated with red meat consumption.

Chronic inflammation is also associated with red meat intake [47], and is a common mechanism for progression of major diseases associated with red meat intake, such as carcinomas [48,49], atherosclerosis [50], and type 2 diabetes [51]. While other mechanisms for chronic inflammation in such diseases are known, Neu5Gc-induced xenosialitis could be one of the missing connections between red meat intake and inflammation-associated disease risk [52]. To assess potential use of anti-Neu5Gc antibodies in large-scale epidemiologic studies, we measured plasma and serum levels in archived longitudinal human samples with well-documented dietary habits: the Nurses’ Health Study II (NHSII), the Health Professionals Follow-up Study (HPFS), and the European Prospective Investigation into Cancer-Norfolk Cohort (EPIC).

First, we used ELISA assays to evaluate human serum or plasma IgGs against simple synthetic Neu5Gc-containing glycan targets and examined correlations of antibody levels against the epitopes with disease risk. Second, we used an ELISA approach against a mixture of natural Neu5Gc-containing epitopes to quantify laboratory variability, stability, and reproducibility of anti-Neu5Gc IgGs over time in the NHS II and HPFS. Third, among women in the NHSII, we also examined correlations between antibody measurements and reported red meat intake. Fourth, we used a sialoglycan array covering >30 distinct Neu5Gc-sialoglycans and their matched Neu5Ac-sialoglycans, to examine the association between anti-Neu5Gc IgG levels and the risk of colorectal cancer among participants in the EPIC-Norfolk Cohort.

## Materials & methods

### Study populations and blood collection

#### a. NHS II and HPFS

The NHS II was established in 1989 among 116,429 female registered nurses, ages 25–42 years. All women completed an initial questionnaire and were followed biennially by mailed questionnaire to update exposure status and disease diagnoses. Data were collected on numerous risk factors including reproductive factors, medical history, family history of cancer, and diet via food frequency questionnaire (see below; Dietary Assessment, section a.). Between 1996 and 1999, 29,611 NHSII cohort members who were cancer-free and between the ages of 32 and 52 years provided blood samples [53]. A subgroup of 304 women in NHSII provided two to three blood samples over a period of 1 to 3 years. For each collection, women had their blood drawn into 10 ml tubes treated with sodium heparin (BD, New Jersey, USA). The HPFS, a parallel cohort of older men, began in 1986 with recruitment of 51,529 male health professionals age 40–75 years, 18,225 of whom donated blood samples between 1993 and 1995 [54]. HPFS participants donated blood in 10 ml tubes treated with ethylenediaminetetraacetic acid (EDTA) (BD, New Jersey, USA). Blood samples from all three cohorts were shipped with an icepack, via overnight couriers, to the Brigham and Women’s Hospital (BWH)/Harvard Cohorts Biorepository, where they were processed into plasma, red blood cells, and buffy coat, and archived in liquid nitrogen freezers (≤-130°C) until aliquots were assayed for anti-Neu5Gc IgG antibodies.

To determine the stability of biomarkers in archived blood specimens with delayed processing due to overnight shipment, blood samples were also collected from 16 healthy community volunteers (i.e., not NHS or HPFS participants). For each volunteer, half of the blood sample was collected in EDTA tubes (three tubes), and the other half was collected in heparin tubes (three tubes). The first of each type of anticoagulant tube was immediately centrifuged at 1530 x g for 20 min at 4°C. After centrifugation, the plasma was removed and aliquots were placed in cryotubes and stored in liquid nitrogen freezers (−130°C). The second and third tubes were shipped with an ice pack to the laboratory overnight and then processed and frozen at 24 h and 48 h, respectively, after blood collection. These processing methods were designed to mimic the collection procedures used for cohort participants. The study was approved by the Committee on the Use of Human Subjects in Research at the Harvard T.H. Chan School of Public Health and the Brigham and Women’s Hospital. All data and samples were fully anonymized prior to access by authors for data analysis.

#### b. EPIC-Norfolk

EPIC began as a multi-centered pan European cohort study examining diet and lifestyle factors and their relation to cancer [55]. EPIC-Norfolk, one of the participating cohorts, is a cohort of men and women aged 40–79 years living in Norfolk UK and recruited from participating general practitioner clinics between 1993 and 1998. A total of 25,639 participants completed a lifestyle questionnaire on recruitment and attended a clinic where non-fasting blood samples were obtained by venipuncture into plain and citrate bottles. After overnight storage in a dark box in a refrigerator at 4–7°C, they were spun at 2,100 x g for 15 min at 4°C, and serum samples were obtained [56]. After processing, samples were stored in freezers at –80°C until laboratory analysis. The EPIC-Norfolk study was approved by the Norfolk and Norfolk Research Ethics Committee. All participants gave signed informed consent for their data and samples to be used in research. All data and samples were fully anonymized prior to access by authors for data analysis.

### Dietary assessment

#### a. NHS II

Every four years since 1991, NHS II participants have filled out a semi-quantitative food frequency questionnaire (FFQ) which included over 130 food items. Participants were asked how frequently they have consumed each food item over the previous year by selecting from nine possible responses ranging from less than once per month to six or more times per day. We calculated total red meat consumption in servings per day as the sum of intakes of individual food items, including unprocessed red meat (beef, pork, or lamb as a sandwich, pork as a main dish, beef or lamb as a main dish, and hamburger) and processed red meat (hot dogs, bacon, and other processed meat such as sausage, salami, bologna). The reproducibility and validity of food frequency questionnaires for measuring diet in the NHS and HPFS cohorts including red meat intake has been documented previously [57–59]. For example, the correlation coefficients for intake of individual red meat items comparing diet records with the FFQ were mostly higher than 0.5 after correction for attenuation due to within person variation between diet records [60].

#### b. EPIC-Norfolk

Within the EPIC-Norfolk cohort, dietary intake was estimated using a 7-day food diary, a structured booklet enabling study participants to record food eaten at different times of the day over a period of one week. Photographs of dishes were included in the diary to help participants estimate the size of portions being consumed [61]. All participants attending the EPIC clinic for venipuncture were also given detailed instructions on how to complete the diary. They were asked to recall the previous day’s intake which was recorded by the interviewer in the diary. The remaining 6 days were completed by the participant at home, and the booklet was returned by post. Food and nutrient intake was calculated using DINER, a bespoke data entry and analysis system [62–64].

### Assays for circulating Anti-Neu5Gc antibodies using different target antigens in ELISA assays

#### a. EPIC-Norfolk cohort samples assayed using Neu5Gcα2-polyacrylamide as the ELISA target

Human serum anti-Neu5Gc IgGs were detected by ELISA as described previously [65]. Briefly, microtiter plates were coated in triplicates with Neu5Acα2-polyacrylamide (PAA) or Neu5Gcα2-PAA (GlycoTech) at 500 ng/well in 50 mM of sodium carbonate-bicarbonate buffer (pH 9.5) at 4°C for overnight. Plates were washed with Tris-buffered saline (TBS) and blocked with Tris-buffered saline with 0.1% Tween 20 (TBST) for 2 hours at room temperature (RT). Dilutions of human serum (1/50 in TBST) were added in triplicates to the wells and incubated for 4 hours at RT. Wells were washed with TBS, and then horseradish peroxidase (HRP)-conjugated goat anti-human IgG (Jackson ImmunoResearch Laboratories) diluted in TBST (1:5000) added to the wells for 1.5 hours at RT. Samples in wells were developed with a buffer containing HRP substrate *O*-phenylenediamine and measured at OD_490 nm_ on a SpectraMax 250 (Molecular Devices). Anti-Neu5Gcα2-PAA IgG values were obtained after subtraction of the values obtained with Neu5Acα2-PAA, thus negating any non-specific binding to Neu5Ac or PAA. Data normalized OD_490 nm_ levels were quantified into μg/ml using standard dilution curves of purified human IgG (Jackson ImmunoResearch Laboratories) per experimental day.

#### b. EPIC-Norfolk cohort samples assayed with Neu5Gcα2–6Galβ1–4Glcβ-human serum albumin (Neu5Gcα2–6Lacβ-HSA) as the ELISA target

The samples that were tested in the above assay were used also for this ELISA target. Briefly, microtiter plates were coated with Neu5Gcα2–6Gal*β*1–4Glc*β*- attached to human serum albumin (Neu5Gcα2–6Lac***β***-HSA) [66] at 1 μg/well in 50 mM sodium carbonate-bicarbonate buffer (pH 9.5) at 4°C for overnight. Each plate was also coated with serial dilutions of purified human IgGs (Jackson ImmunoResearch Laboratories; 10–0.3 ng/well) in the same buffer. Wells were blocked for 2 hours at RT with 1% ovalbumin (Grade V, Sigma, free of Neu5Gc) in PBS, followed by incubation with serum samples diluted 1/100 in the same blocking solution for 2 hours at RT. The plates were washed three times with PBS containing 0.1% Tween (PBST) by a plate washer (Molecular Devices Microplate Skanwasher) and subsequently incubated for 1 hour at RT with 1/100 diluted (5 ng/μl) phycoerythrin-conjugated anti-human IgG (Jackson ImmunoResearch Laboratories; R-Phycoerythrin AffiniPure goat anti-human IgG, Fcγ Fragment Specific #109-115-098). After washing three times with PBST, wells were filled with 100 μl of PBS and samples were read on a fluorescence plate scanner. Neu5Gc-specific antibody levels were defined by subtracting the readings obtained with the Neu5Gcα2–6Lac***β***-HSA target from the readings obtained with the Neu5Acα2–6Lac***β***-HSA HSA target. Fluorescence values were quantified into μg/ml by comparisons with a standard dilution curve of purified human IgGs coated on the same ELISA plate.

#### c. EPIC-Norfolk and NHS-II cohort samples assayed using naturally occurring Neu5Gc-glycans in wild-type mouse sera as the ELISA target

Microtiter plate (Costar 9018) wells were coated overnight at 4°C with 1 μg/well of this Neu5Gc-rich wild-type mouse sera containing diverse Neu5Gc-sialoglycoproteins (as previously described in [49, 50] that lacked mouse-anti-human reactivity in coating buffer (50 mM sodium carbonate-bicarbonate buffer, pH 9.5). Serial dilutions of human IgG (10–0.3 ng/well) were also coated in the same plate for quantification. Wells were blocked with PBS-Ova (PBS pH 7.4, 1% chicken ovalbumin) for 1 hour at RT. During the blocking step, the human samples (plasma for the NHS-II samples and serum for the EPIC-Norfolk samples) were diluted 1/100 in PBS-Ova containing 1:4000 *Cmah*^-/-^ pooled sera, in order to block non-Neu5Gc binding to the target, as previously described [67]. To evaluate specificity, another set of human samples were prepared as above but with the addition of 5 mM methyl-α-Neu5Gc (Neu5Gcα2Me) [20] for inhibition. Diluted human samples (100 μl each) were added to wells in triplicate and incubated for 2 hours at RT. Wells were then washed 5 times with PBST (PBS pH 7.4, 0.05% Tween-20) by a microplate washer (Molecular Devices Microplate Skanwasher) followed by detection with 100 μl/well HRP-conjugated goat anti-human IgG (Bio-Rad #172–1050, 1:7000 dilution) in PBS for 1 hour at RT. After washing 5 times with PBST as above, the HRP substrate *O*-phenylenediamine was added and the signal was developed for 20 minutes before the reaction was terminated with H_2_SO_4_. Absorbance was measured at 490 nm on SpectraMax M3 (Molecular Devices). The signals from the second set of human samples prepared with Neu5Gcα2Me were subtracted from the signals obtained by the first set prepared without Neu5Gcα2Me. Absorbance was interpolated using the standard curve generated by the human IgG wells. Thus, we measured antibody titers for both “total” anti-Neu5Gc IgG and “inhibitable” anti-Neu5Gc IgG.

#### d. Sialoglycan microarray assays using multiple specific Neu5Gc-glycans

The anti-Neu5Gc IgG responses to 31 pairs of Neu5Ac and Neu5Gc-terminated glycans (S1 Table) were generated as Relative Fluorescence Units (RFU). The sialoglycan pairs (see results for details) were synthesized as previously described [68] and printed on Epoxide slides (Thermo Fisher Scientific, Corning, Pittsburgh, PA) in 100 μM at 4 replicates each in an optimized print buffer (300 mM phosphate buffer, pH 8.4). Printed glycan microarray slides were blocked with 0.05 M ethanolamine in Tris-HCl (0.1 M, pH 9.0), washed and dried. Slides were fitted in a multi-well microarray hybridization cassette (AHC4X8S, ArrayIt, Sunnyvale, CA, USA) to divide into 8 subarrays. The subarrays were blocked with ovalbumin (1% w/v) in PBS (pH 7.4) at RT for 1 h, with gentle shaking. Subsequently, the blocking solution was removed and diluted serum samples with 1/100 dilution were added to each subarray. After incubating the samples at RT for 2 hours with gentle shaking, the slides were washed. Goat anti-human IgG-Cy3 at 1.5 μg/ml antibody (Jackson ImmunoResearch Laboratories) in PBS was added to the subarrays, incubated for 1 hour at RT, washed and dried. The microarray slides were then scanned by Genepix 4000B microarray scanner (Molecular Devices Corp., Union City, CA, USA) and data analysis was performed using Genepix Pro 7.0 analysis software (Molecular Devices Corp., Union City, CA). Although each of the 31 pairs of Neu5Ac- and Neu5Gc-terminated glycans generated an antibody response measured as a Relative Fluorescence Unit (RFU), the sum total against all Neu5Gc-terminated glycans was also used for statistical analyses.

### Case-control studies using EPIC samples

a. Coronary heart disease. EPIC plasma samples (citrate) were examined from 858 individuals who were diagnosed with coronary artery disease (CAD) up to 8.5 years after they donated blood and 1869 sex and age matched CAD-free controls. CAD cases with prevalent self-reported heart attack or stroke at baseline and those with no available plasma (citrate) were excluded leaving plasma samples on 835 cases for analysis.

b. Colorectal cancer. Another case-control study of EPIC-Norfolk cohort plasma samples consisting of cancer cases from any site (except non-melanoma skin cancer), who were matched to controls by sex, age at blood donation (within 5 years) and recruitment date (within 3 months) was also established. Cases were defined by ICD codes I20-I25 appearing on a death certificate or on hospital discharge records. Participants who reported a history of cancer at recruitment were not eligible as cases or controls. Of the 493 total cancer cases diagnosed up to 19.8 years after blood donation (mean 18.6 years), 71 colorectal cancer cases and matched controls were included for this analysis. Samples from cases and controls were mixed and arranged randomly in boxes for shipping such that the receiving laboratory was blind to case-status.

### Statistical analyses

#### a. Anti-Neu5Gc IgG inter-assay reproducibility, stability and within-person reproducibility in NHS II and HPFS

The Results Section summarizes our three analyses to assess anti-Neu5Gc IgG inter-assay reproducibility, stability with delayed sample processing, and within-person reproducibility over 1–3 years in archived human plasma samples (Table 1) In experiment #1, the split pilot study, we measured anti-Neu5Gc IgG antibodies in 18 blinded duplicate plasma samples donated by participants in the NHS and HPFS and 3 quality control (QC) pool plasma replicates. We noted a discontinuous distribution and categorized anti-Neu5Gc IgG levels in four groups based on the observed overall distribution (cut-points were at the 10^th^ percentile, 75^th^ percentile, and at 10 ng/dl). We assessed inter-assay reproducibility among the split samples by calculating Spearman correlation coefficients, as well as by calculating the number of concordant vs. discordant pairs based on the categories described above and the associated Kappa statistic to measure agreement in classification. Assay reproducibility over a 24–48 hours processing delay (n = 48 samples; 3 samples each from 16 individual donors; experiment #2), and within-person reproducibility over 1–3 years (n = 94 samples; 2 samples each from 47 NHSII participants; experiment #3), were assessed using Spearman and intra-class correlation coefficients (ICCs). The median time between the two blood samples for the reproducibility study was 23 months with a range of 10 to 32 months [69]. No differences in were seen in heparin vs. EDTA plasma (data not shown); therefore, combined results are presented.

**Table 1:** Description of experiments for inter-assay reproducibility, stability with processing delays, and short-term within-person stability.

Experiment	1. Inter-assay reproducibility	2. Stability with processing delays	3. Short-term within-person reproducibility
Participants	NHS & HPFS; QC pools	Healthy volunteers	NHS II
N	21	16	47
Sample	Heparin & EDTA plasma	Heparin & EDTA plasma	Heparin plasma
Number of aliquots	2	3	2
Timing	Split sample	(1) Processed immediately (2) Stored 24 h at 4°C (3) Stored 48 h at 4°C	1–3 years
Storage conditions	Liquid nitrogen (below -130°C) for 1–2 years	Liquid nitrogen (below -130°C) for 4–5 years	Liquid nitrogen (below -130°C) for 13–14 years

NHS = Nurses’ Health Study; HPFS = Health Professionals Follow-up Study; QC = Quality Control

#### b. Correlation between anti-Neu5Gc IgG antibodies and red meat consumption in NHS II

To assess correlation between measured anti-Neu5Gc IgG antibodies and red meat consumption, we selected a random sample of 338 women in NHSII (representing a wide variety of red meat intake levels), who had provided blood samples and completed the 1999 food frequency questionnaire (FFQ) [69]. We used the 1999 FFQ for this analysis because it was the closest in time to collection of blood samples. Two individuals were missing data on at least one red meat item and were excluded, leaving 338 women for the analysis. The median time from blood draw until the completion of the 1999 FFQ was 8 months; the timing ranged from blood draw 34 months prior to the FFQ to blood draw 6 months after the FFQ [69]. We evaluated the association between anti-Neu5Gc IgG titers with usual adult red meat intake (0, <0.5, 0.5–<1, 1–<2, or 2+ servings per day). We calculated Spearman correlation coefficients and fit a linear regression model to the data, with antibody levels as the dependent variable and categories of red meat intake as the independent variable. The software used for NHS pilot studies and correlation with red meat intake was SAS 9.3 (Cary, NC).

#### c. Case-control studies (EPIC-Norfolk)

**Coronary artery disease**.

Using data from EPIC-Norfolk conditional logistic regression was used to examine 835 coronary artery disease cases and 1869 controls matched for age and sex as described above. Each of the four analytes was divided into quintiles with quintile 1 (the lowest) used as the reference. Models adjusting for just age and sex and for multiple variables were performed. Multivariable models adjusted for age, sex, body mass index, total cholesterol (measured using an RA 1000 Technicon analyzer (Bayer Diagnostics, Basingstoke) from non-fasting blood samples taken by venipuncture), systolic blood pressure (mean of two readings), smoking status (never, former, current) and diabetes mellitus (self-reported).

**Colorectal cancer**.

Similarly, we fitted conditional logistic regression models to estimate odds ratios and 95% confidence intervals (CIs) for risk of colorectal cancer, associated with quartiles of total anti-Neu5Gc IgG antibodies (the sum of RFU values of antibodies against all Neu5Gc-glycans on the array), with quartile 1 (the lowest) used as the reference. Model 1 was unadjusted (i.e., just adjusted for matching factors) while model 2 was adjusted for body mass index (quintiles), height (quintiles), smoking status (never, former, current), physical activity (four categories from low to high based on occupational and leisure time physical activity) [70] aspirin use over previous three months (yes or no), education level (no qualifications or some qualifications), occupational social class (manual or non-manual), and family history of cancer in a first degree relative (yes or no). To test for linear trend, we modeled the quartiles as an ordinal variable. However, since the anti-Neu5Gc IgG antibodies were highly skewed, we also modeled the natural log of the sum of glycans as a continuous variable. Linear regression was used to investigate the relationship between red meat intake and natural log of the sum of glycans in cases and controls separately. Two-sided p-values <0.05 were considered statistically significant. The software used for EPIC-Norfolk analyses were Stata v.14 (StataCorp, College Station, Texas, USA) and R v.3 with packages ggplot2,corrplot,dplyr (R Foundation, Vienna, Austria).

## Results

### Levels of antibodies against simple defined Neu5Gc-containing epitopes are not associated with coronary artery disease risk among the EPIC-Norfolk cohort

**Fig 1:**
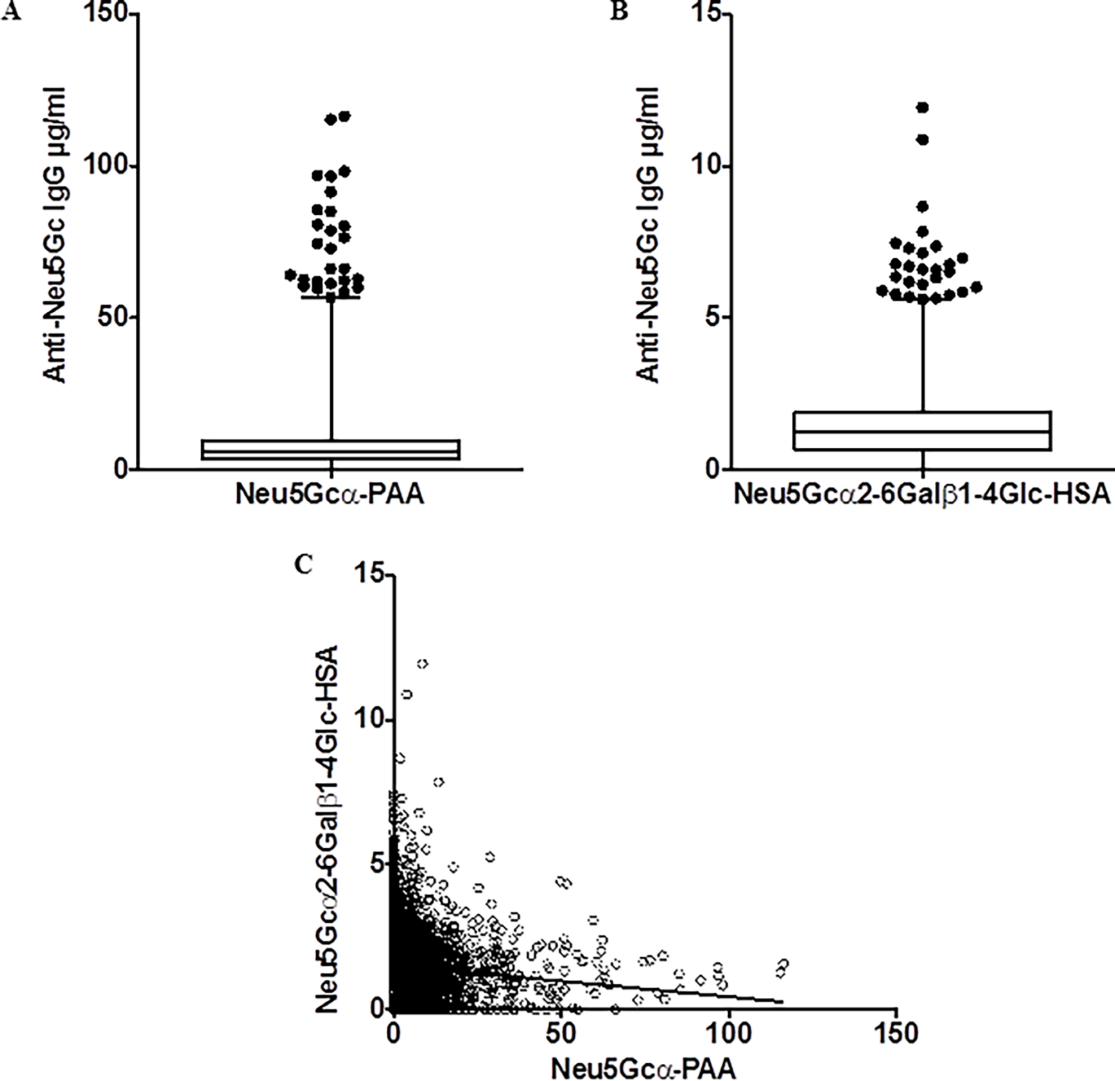
Distribution of Anti-Neu5Gc IgG titers in EPIC-Norfolk cohort using defined ELISA target antigens. Each dot represents a value for a single subject. (A) Levels of serum anti-Neu5Gc IgG quantified by ELISA using Neu5Gcα2-polyacrylamide (Neu5Gcα2-PAA) as the target antigen (N = 2716). The mean anti-Neu5Gc IgG titer was 8.6 μg/ml (SD±10.6). (B) Levels of serum anti-Neu5Gc IgG quantified by ELISA using Neu5Gcα2–6Lacβ-human serum albumin (HSA) as the target antigen (N = 2712). The mean anti-Neu5Gc IgG titer was 1.4 μg/ml (SD±1.2). (C) There was no correlation between anti-Neu5Gc IgG antibody levels directed against the two different chemically synthesized ELISA targets: Neu5Gcα2-PAA and Neu5Gcα2–6Lacβ-HSA.

It was originally thought that circulating antibodies against Neu5Gc-glycans were only found in patients with cancer or other chronic inflammatory diseases [7]. However, using a more sensitive ELISA assay, we subsequently discovered that all individuals tested had some levels of detectable anti-Neu5Gc antibodies against a synthetic polyacrylamide (PAA) backbone presenting multiple copies of the simple monosaccharide epitope Neu5Gcα2- (Neu5Gcα2-PAA), when compared with the human sialic acid counterpart Neu5Acα2-PAA, which differs by a single oxygen atom [9]. This assay was used to study an EPIC population of incident coronary artery disease and age and sex matched controls. The anti-Neu5Gcα2-PAA antibody levels showed a highly skewed non-Gaussian distribution in the population (Fig 1A), but there was no significant association with CAD risk (Tables 2 and 3 and S4 Table).

**Table 2:** Comparison of serum anti-Neu5Gc antibody levels between coronary artery disease cases and controls matched for age and sex in the EPIC-Norfolk cohort.

	Controls (N = 1869)	Cases (N = 835)
	**Mean (SD)**	
Anti-Neu5Gc IgG against Neu5Gc-alpha-PAA (μg/mL)	13.74 (13.24)	12.96 (10.51)
Anti-NeuGc IgG against Neu5Gc2-6Lac-HSA (μg/mL)	7.53 (3.08)	7.18 (2.66)
Total anti-Neu5Gc IgG against mouse serum Neu5Gc-terminated glycans (μg/mL)^a^	1.73 (1.68)	1.57 (1.29)
Neu5Gc Inhibitable IgG against mouse serum Neu5Gc-terminated glycans (μg/mL)^a^	0.43 (1.26)	0.36 (1.07)
Age (years)	65.3 (7.7)	65.6(7.7)
BMI (kg/m2)	26.3 (3.5)	27.3 (3.8)
Systolic blood pressure (mm Hg)	138.7 (17.6)	143.4 (18.6)
Cholesterol (mmol/l)	6.28(1.17)	6.51(1.25)
LDL-cholesterol (mmol/l)	4.06 (1.00)	4.26 (1.05)
HDL-cholesterol (mmol/l)	1.39 (0.41)	1.27 (0.36)
White cell count	6.5 (1.7)	7.0 (2.1)
C-Reactive protein	3.27 (5.76)	4.76 (7.78)
	**% (n)**	
Men	62 (1157)	62 (521)
Current smokers	8.4 (156)	15.1 (125)
Diabetes mellitus history	1.9 (35)	6.6 (55)

^a^Data available for 1076 controls and 386 cases

**Table 3:** Odds ratios for coronary heart disease by quintile of each analyte for 835 coronary artery disease cases and 1869 controls in the EPIC-Norfolk cohort.

	Quintile 1	Quintile 2	Quintile 3	Quintile 4	Quintile 5
Anti-Neu5Gc IgG against Neu5Gc-alpha-PAA (μg/mL)	<6.38	6.38–8.89	8.90–11.63	11.64–16.84	16.86+
Age and sex adjusted	1	1.14 (0.88–1.49)	1.21 (0.93–1.57)	1.15 (0.89–1.50)	0.99 (0.76–1.30)
Multivariate adjusted^b^	1	1.15 (0.87–1.51)	1.18 (0.90–1.55)	1.18 (0.90–1.55)	0.99 (0.75–1.31)
Anti-Neu5Gc IgG against Neu5Gc2-6Lac-HSA (μg/mL)	<5.29	5.29–6.23	6.24–7.16	7.17–8.87	8.88+
Age and sex adjusted	1	1.09 (0.85–1.41)	1.18 (0.81–1.52)	1.03 (0.80–1.33)	0.74 (0.56–0.96)
Multivariate adjusted^b^	1	1.13 (0.86–1.47)	1.28 (0.98–1.68)	1.07 (0.82–1.40)	0.81 (0.61–1.08)
Total anti-Neu5Gc IgG against mouse serum Neu5Gc-terminated glycans (μg/mL)^a^	<0.81	0.81–1.10	1.11–1.46	1.47–2.08	2.09+
Age and sex adjusted	1	1.06 (0.73–1.53)	1.31 (0.91–1.88)	1.13 (0.78–1.64)	0.89 (0.60–1.30)
Multivariate adjusted^b^	1	0.97 (0.65–1.43)	1.34 (0.91–1.97)	1.15 0.78–1.69)	0.95 (0.64–1.42)
Neu5Gc Inhibitable IgG against mouse serum Neu5Gc-terminated glycans (μg/mL)^a^	<-0.05	-0.078	0.029–0.129	0.130–0.481	0.482+
Age and sex adjusted	1	0.88 (0.61–1,27)	1.03 (0.72–1.48)	0.86 (0.60–1.25)	0.83 (0.57–1.19)
Multivariate adjusted^b^	1	0.91 (0.62–1.34)	1.05 (0.72–1.52)	0.84 (0.57–1.24)	0.91 (0.62–1.34)

^a^ Data available for 1076 controls and 386 cases

^b^ Adjusted for age, sex, body mass index, total cholesterol, systolic blood pressure, smoking status and diabetes mellitus

We reasoned that such antibodies against a single monosaccharide might not be as specific because the binding pockets of antibodies typically accommodate 3 to 5 monosaccharides [71,72]. Based on our prior work showing that antibodies against the more extended epitope Neu5Gcα2–6Lacβ–R were relatively common in the population [20], we next tried an ELISA assay against chemically synthesized Neu5Gcα2–6Lacβ-HSA. We again found a skewed non-Gaussian distribution of levels in the population (Fig 1B), but no significant association with CAD risk (Tables 2 and 3 and S4 Table), similar to the results with Neu5Gcα2-PAA.

Moreover, as shown in Fig 1C, there was no significant correlation between the levels of antibody directed against these two epitopes (Neu5Gcα2-PAA and Neu5Gcα2–6Lacβ-R), suggesting that these two assays were detecting different populations of circulating antibodies (Spearman correlation co-efficient is -0.1363 and the p-value is <0.0001). It should also be noted that the majority of the sera showed IgGs at just detectable levels in both assays, consistent with a recent report by others who also “found minimal antibody titer directed against Neu5Gcα and the trisaccharide Neu5Gcα2–6Galβ1–4GlcNAcβ-”, using somewhat similar assays [73].

Measuring antibodies against a more complex mixture of natural Neu5Gc-containing epitopes among EPIC-Norfolk and NHS II cohorts. We reasoned that the lack of association with disease risk may be related to the fact that individual humans have a widely disparate spectrum of antibodies against various Neu5Gc-containing epitopes [20]. To obtain a composite measurement of antibodies against multiple Neu5Gc-containing epitopes we turned to the use of wild-type mouse serum as an ELISA target, since it displays multiple types of Neu5Gc-containing *N*-glycans and *O*-glycans on glycoproteins [67,68,74]. In this instance, we established background binding using serum from congenic *Cmah*^*-/-*^ Neu5Gc-deficient mice. To adsorb antibodies in some human sera directed against unrelated mouse epitopes, we also added *Cmah*^*-/-*^ serum into the fluid phase before and during the first binding step of the ELISA assay [67]. For evaluating specificity of binding, a parallel set of human sera was prepared in a similar way, but with the addition of methyl-α-Neu5Gc (Neu5Gc2Me) for specific inhibition. To calculate the “specific” titer, the signal from the second set incubated with Neu5Gc2Me was subtracted from the signal obtained by the first set without Neu5Gc2Me. Absorbance was interpolated using standard curves generated with human IgG. The readout for “inhibitable” anti-Neu5Gc IgG should theoretically represent the more specific but less sensitive titer (since the incubation step with the free Neu5Gc2Me only blocks some of the specific binding). Negative values may be generated since some individuals could possess cross-reacting anti-Neu5Ac antibodies. Before proceeding further with using this assay we addressed inter-assay reproducibility, stability with delayed processing, and within-person reproducibility over time using the NHS cohort.

### Anti-Neu5Gc inter-assay reproducibility (NHS II cohort)

**Fig 2:**
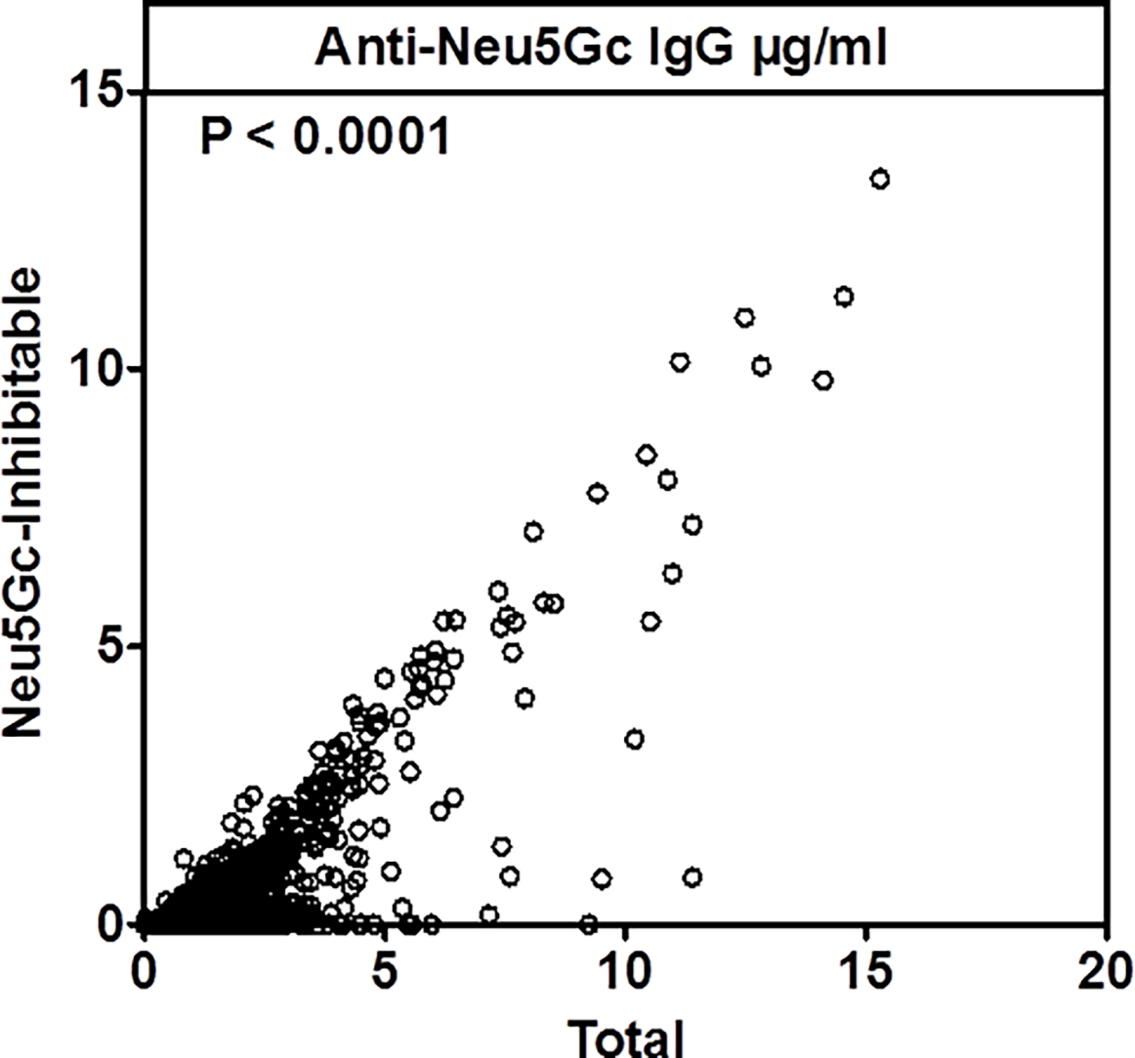
Correlation between total and inhibitable anti-Neu5Gc IgG titers in the NHS cohort. Plasma anti-Neu5Gc IgG levels were quantified by ELISA using wild type mouse serum that contains naturally occurring Neu5Gc-containing epitopes as the target antigen (N = 46). A strong correlation was observed between total and inhibitable anti-Neu5Gc IgG titers in the NHS cohort (Spearman r = 0.80).

We observed good inter-assay reproducibility between blinded split samples for individuals (Table 4A) Spearman correlation coefficients were 0.93 and 0.86 for total and inhibitable anti-Neu5Gc antibody titers, respectively (Fig 2). Considering the categorical variable, 18 out of 21 paired samples were perfectly concordant for total anti-Neu5Gc IgG (weighted Kappa = 0.82) and 15 out of 21 paired samples were perfectly concordant for inhibitable anti-Neu5Gc IgG (weighted Kappa = 0.71) (Table 4A), that together suggest very high inter-assay reproducibility.

**Table 4:** Inter-assay reproducibility, effects of processing delay and within-person reproducibility in the NHS cohort.

**A. Inter-assay reproducibility (n = 21)**	**Total** **anti-Neu5Gc IgG**	**Inhibitable** **anti-Neu5Gc IgG**
Median (minimum-maximum), ng/μL	2.75 (1.27, 21.61)	1.23 (-0.04, 18.26)
Spearman correlation between split pairs:	0.93	0.86
# concordant/discordant pairs (4 categories):	18/3	16/5
Weighted Kappa:	0.82	0.71
**B. Effects of Processing delay (n = 53)**		
Median (minimum-maximum), ng/μL	2.39 (0.49, 7.42)	1.40 (-0.73, 6.24)
Spearman correlation between 0–24 hrs:	0.43	0.65
Spearman correlation between 0–48 hrs:	0.42	0.59
ICC across processing delays:	0.58	0.80
**C. Within-person reproducibility (n = 47)**		
Median (minimum-maximum), ng/μL	1.92 (-0.08, 33.09)	0.10 (-3.33, 29.48))
Spearman correlation between 2 blood draws:	0.84	0.78
ICC across blood draws:	0.94	0.87
# concordant/discordant pairs (4 categories):	38/9	33/14
Weighted Kappa:	0.70	0.62

### Anti-Neu5Gc IgG antibodies demonstrate stability with delayed processing of whole blood samples (NHS II cohort)

Delays in processing of up to 48 hours appeared to have little influence on measurement of plasma anti-Neu5Gc IgG. The overall ICCs across processing delays up to 48 hours were 0.58 and 0.80 for total and inhibitable anti-Neu5Gc IgG, respectively (Table 4B). The Spearman correlation coefficients between samples processed immediately versus after 24 hours were respectively 0.43 for total and 0.65 for inhibitable anti-Neu5Gc IgG antibodies, and were similar for immediately versus after 48 hours (0.42 and 0.59). Using 4 categories of plasma anti-Neu5Gc IgG based on the distribution observed in the first pilot experiment, for immediate versus 24-hour processing, there were 13 out of 16 concordant pairs for total and 14 out of 16 for inhibitable anti-Neu5Gc antibodies. These results indicate that measurement of this marker is feasible, particularly the inhibitable anti-Neu5Gc IgG antibodies, in large-scale epidemiologic studies.

### Anti-Neu5Gc IgG antibody levels are stable over time (NHS II cohort)

Within a subset of women in the NHSII who provided repeated blood samples (n = 47), anti-Neu5Gc IgG antibodies remained stable over a time period of 1 to 3 years from the prior anti-Neu5Gc level (Table 4C). The Spearman correlation coefficients between blood draws were 0.84 and 0.78 for total and inhibitable anti-Neu5Gc antibody titers, respectively, and the ICCs were 0.94 and 0.87, respectively. Based on four categories, we noted 38 concordant pairs out of 47 total pairs for total anti-Neu5Gc IgG (weighted Kappa = 0.70) and 33 concordant pairs out of 47 total pairs (weighted Kappa = 0.62) for inhibitable anti-Neu5Gc IgG.

In summary, the above results indicate that this ELISA measurement of multiple anti-Neu5Gc IgG antibodies in blood samples is reliable, with low laboratory errors, stable in samples with delayed processing up to 48 hours, and strongly reproducible within subjects over time—providing evidence that plasma anti-Neu5Gc IgG can be used in large epidemiologic studies involving stored samples.

### Distribution of Anti-Neu5Gc IgG in the NHS II and EPIC cohorts

**Fig 3:**
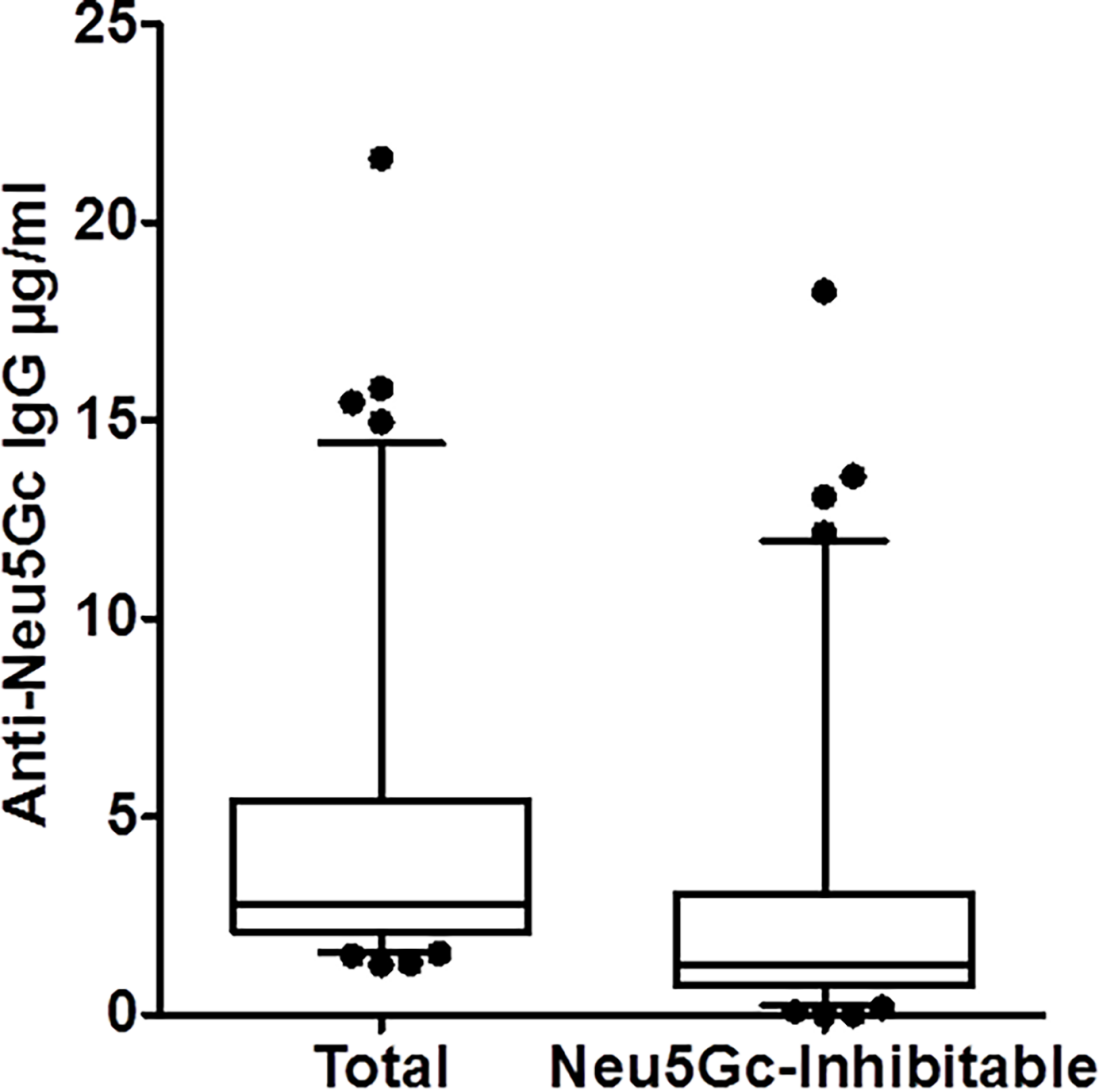
Distribution of anti-Neu5Gc IgG titers against a mixture of epitopes in NHS II population. Plasma total and inhibitable anti-Neu5Gc IgG levels were quantified by ELISA using wild-type mouse serum as a target that contains naturally occurring Neu5Gc containing sialoglycoproteins epitopes as antigens (N = 46; Table 1). Total anti-Neu5Gc IgG refers to the “raw” antibody level obtained against the ELISA target. To obtain a more specific titer, plasma was incubated with free Neu5Gcα2Me to block non-specific binding and the inhibitable anti-Neu5Gc IgG is this signal subtracted from the total signal. Plasma anti-Neu5Gc IgG antibodies in the NHS II population displayed a discontinuous distribution. While the majority of individuals have low levels, a minority of samples (represented by dots) exhibit very high levels of anti-Neu5Gc IgG.

**Fig 4:**
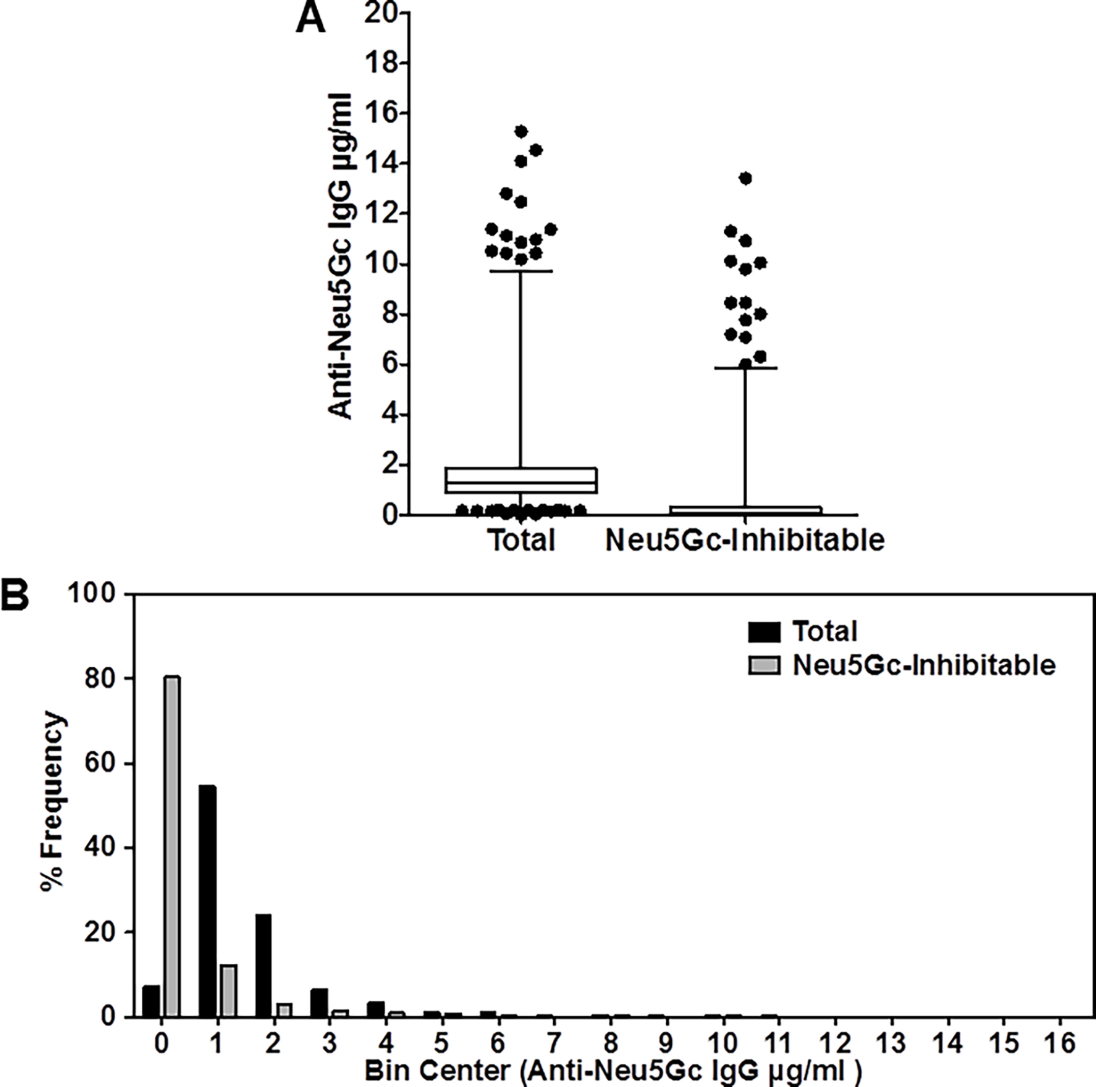
Distribution of Anti-Neu5Gc IgG titers against a mixture of epitopes in the EPIC-Norfolk cohort. (A) Serum anti-Neu5Gc IgG antibodies in the EPIC-Norfolk population assayed as in Fig 1. Serum total and inhibitable anti-Neu5Gc IgG levels were quantified by ELISA using wild type mouse serum that contains naturally occurring Neu5Gc containing epitopes as the target antigen (N = 1469). The mean total IgG was 1.7 μg/ml (SD±1.6) and the mean inhibitable IgG was 0.4 μg/ml (SD±1.2). Total and “inhibitable” anti-Neu5Gc IgG were defined as in Fig 1. (B) The distribution of levels of anti-Neu5Gc IgG was evaluated by dividing all the samples into 16 bins, in steps of 1 μg/ml each, and the frequency of number of tested samples in each bin described as bar charts for both total (black) and inhibitable (grey) IgG values.

The target antigen for the ELISA assay was wild-type mouse serum discussed above, which exhibits a limited range of naturally occurring Neu5Gc-containing sialoglycoproteins epitopes. Among the 46 samples (18 individuals plus 3 QC pools) included in the split pilot study, plasma anti-Neu5Gc IgGs displayed a discontinuous skewed distribution, with few samples exhibiting high values (>10 μg/ml) and the majority having low levels of anti-Neu5Gc IgG antibodies (Fig 3). There was strong correlation between total and inhibitable anti-Neu5Gc IgG antibodies (Spearman r = 0.80) (Fig 2). A similar discontinuous distribution was identified in the EPIC study population as well (Fig 4A and 4B), with the inhibitable IgG showing lower values compared to the measured total anti-Neu5Gc IgG (Figs 3 and 4B). Yet similar to the previous analysis on the single Neu5Gc-epitopes, measured anti-Neu5Gc IgG was not associated with CAD risk (Tables 1 and 2 and S4 Table).

### Anti-Neu5Gc IgG levels did not correlate with red meat intake within the NHS II cohort

**Fig 5:**
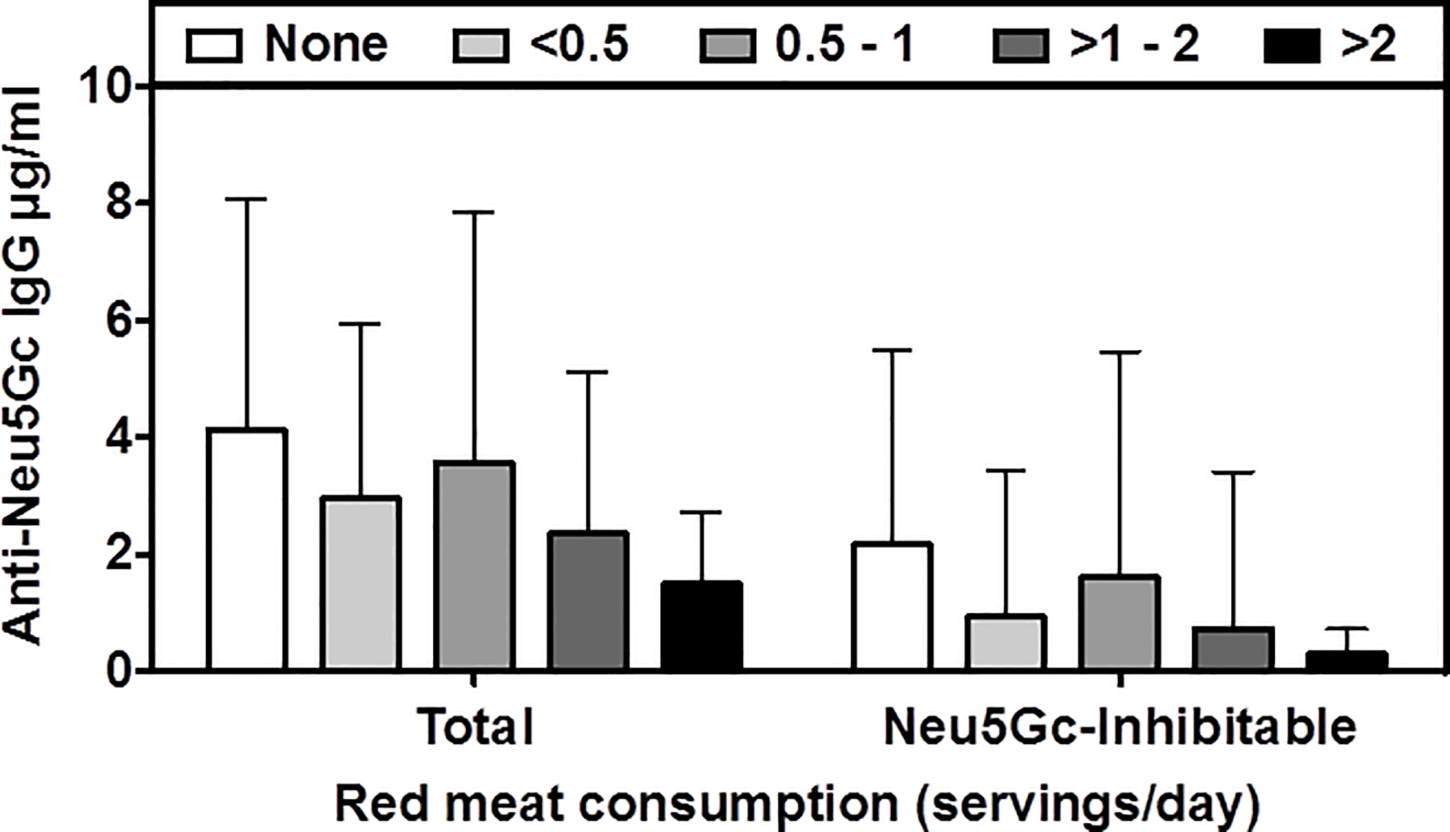
Lack of Correlation between Anti-Neu5Gc IgG titers and red meat intake in the NHS II population. Plasma anti-Neu5Gc IgG levels (total anti-Neu5Gc IgG levels, or inhibitable anti-Neu5Gc IgG levels) were quantified by ELISA using wild type mouse serum as a target that contains multiple naturally occurring Neu5Gc containing epitopes (N = 338). Individuals were sorted into categories based on red meat servings/day (s/d).

Red meat consumption leads to metabolic incorporation of Neu5Gc, generating xeno-auto-glycans that could be involved in tumor stimulation via chronic inflammation. In the human-like *Cmah*^-/-^ mice, Neu5Gc-fed mice demonstrated a five-fold increase in the carcinoma incidence in the liver, an organ that accumulates Neu5Gc in mice [75]. However, while tumor growth was stimulated at low antibody doses, it was inhibited by much higher doses [68] over a linear range [76]. Thus, it is essential to examine the factors influencing circulating levels of anti-Neu5Gc. Given that the major source of human antigen exposure is dietary red meat, we evaluated whether red meat consumption was correlated with plasma antibody levels. Among 338 individuals in the NHS II with available FFQ information, we did not observe higher plasma anti-Neu5Gc IgG titers with increasing red meat intake (Spearman correlation coefficients were -0.05 and -0.02 for total and inhibitable anti-Neu5Gc, respectively). Among women who reported no red meat consumption (n = 15), mean total anti-Neu5Gc IgG antibody level was 4.1 μg/ml, whereas among women who consumed >2 servings/day of red meat (n = 6), mean antibody level was 1.5 μg/ml (Fig 5). Further, there was no evidence of a significant association in regression analyses (p-values of 0.13 and 0.18 for total and inhibitable anti-Neu5Gc IgG, respectively). Similarly, no associations were observed when considering processed and unprocessed red meat products separately, or when considering the cumulative average intake from baseline through 1999 (data not shown). These data are consistent with our earlier data suggesting that spontaneous immunization against Neu5Gc glycans may occur via uptake and presentation of dietary Neu5Gc by bacteria like *H*. *influenzae* early in life [21] may be a more important determinant of subsequent levels during adult life.

### Combined total anti-Neu5Gc IgG levels determined by sialoglycan array are associated with colorectal cancer risk in the EPIC-Norfolk cohort

Given that Neu5Gc can cap a variety of different underlying glycans, the immune response against it is diverse and polyclonal (despite Neu5Gc being the terminal antigenic epitope) [20]. Thus, while the ELISA assay described using the mouse serum target provides a general overview of the anti-Neu5Gc IgG response and maybe tested in future as a screening tool, it does not cover all the common Neu5Gc-containing epitopes that would be found on epithelial or endothelial surfaces *in vivo*. A much broader view of specific antibody response against precise Neu5Gc-containing glycan can be obtained using a sialoglycan microarray that displays most of the common naturally-occurring Neu5Gc-containing epitopes [77]. Such data could potentially be useful for identifying a specific anti-Neu5Gc antibody for a particular disease process. On the other hand, this microarray approach is far more expensive and labor intensive, so a smaller study was set up to assess its value, targeting colorectal cancer, a risk which has been best established for red meat consumption.

We analyzed the anti-Neu5Gc IgG profiles for 71 colorectal cases and 71 age and sex-matched controls within the EPIC-Norfolk cohort, using a custom sialoglycan array that includes 31 pairs of Neu5Ac/Neu5Gc-terminated glycans (S1 Table). When evaluating antibodies directed against these individual Neu5Gc-containing epitopes on the array (S2 Table), we did not observe any strong association between CRC risk and any of the epitopes, including antibody levels against Neu5Gcα2–6GalNAcαOR (GcSTn), which we had previously found to be more prominent in patients with carcinomas than with other diseases [68].

Since antibody levels against any single Neu5Gc-glycan were not predictive of risk, we considered the combined total (sum) of all anti-Neu5Gc-glycan IgG antibody levels, as measured on the array as Relative Fluorescence Units (RFUs). Table 5 demonstrates this sum of all anti-Neu5Gc-glycan IgG antibody RFU levels (“total antibodies”) measured on the array, divided into quartiles. As with the ELISA results, the discontinuous skewed distribution is notable. S3 Table shows the descriptive characteristics by case control status. By design, cases and controls were similar with respect to age and sex distribution. They were also similar with regard to other factors, such as body mass index, physical activity, smoking history, aspirin use, blood pressure, occupation, education level, family history of cancer, and red meat intake. Controls had slightly higher total cholesterol and slightly greater intakes of total energy, total fat and dairy compared to cases, while cases had slightly higher alcohol consumption than controls. S2 Table shows the mean, standard deviation, median, and inter quartile range of the individual glycans by case control status.

**Table 5:** Descriptive characteristics of the colorectal cancer case-control study for anti-Neu5Gc IgG levels, divided into quartiles in the EPIC-Norfolk cohort.

	All	Quartile 1 (n = 36; 25.4%)	Quartile 2 (n = 35; 24.6%)	Quartile 3 (n = 36; 25.4%)	Quartile 4 (n = 35; 24.6%)
**Sum of antibodies against all Neu5Gc-glycans**					
Mean ± SD	6845.2 ± 18163.7	453.8 ± 287.2	1438.4 ± 277.5	2917.5 ± 812.7	22866.0 ± 31834.8
**Sum of antibodies against all Neu5Gc-glycans (median)**					
Median (IQR)	1928.0 (933.9–4554.8)	383.2 (255.8–708.1)	1393.5 (1234.2–1678.9)	2573.5 (2266.6–3544.8)	9102.0 (6750.9–24271.8)
**Inhibitable Anti-Neu5Gc IgG**					
Mean ± SD	0.4 ± 1.3	0.1 ± 0.4	0.1 ± 0.3	0.3 ± 0.8	1.2 ± 2.3
**Total Anti-Neu5Gc IgG**					
Mean ± SD	1.7 ± 1.7	1.6 ± 1.6	1.3 ± 0.8	1.6 ± 1.0	2.5 ± 2.6
**Age, years**					
Mean ± SD	67.1 ± 6.1	67.2 ± 5.2	66.8 ± 7.0	66.5 ± 6.0	67.8 ± 6.1
**Sex**					
Men (%)	98 (69.0)	24 (66.7)	26 (74.3)	26 (72.2)	22 (62.9)
Women (%)	44 (31.0)	12 (33.3)	9 (25.7)	10 (27.8)	13 (37.1)
**Body mass index, kg/m^2^**					
Mean ± SD	26.6 ± 3.4	26.9 ± 3.2	26.3 ± 2.8	27.6 ± 4.2	25.7 ± 3.0
**Weight, kg**					
Mean ± SD	76.6 ± 12.3	75.9 ± 12.4	77.5 ± 11.6	79.8 ± 13.1	72.9 ± 11.4
**Height, cm**					
Mean ± SD	169.3 ± 8.9	167.7 ± 9.6	171.3 ± 10.1	170.2 ± 8.2	168.0 ± 7.4
**Units of Alcohol per week**					
Mean ± SD	7.8 ± 8.5	8.4 ± 8.7	6.1 ± 7.7	8.5 ± 8.1	8.3 ± 9.4
**Total cholesterol**					
Mean ± SD	6.4 ± 1.2	6.4 ± 1.2	6.4 ± 1.3	6.4 ± 1.1	6.3 ± 1.4
**Systolic blood pressure**					
Mean ± SD	137.8 ± 18.1	140.5 ± 20.3	137.3 ± 17.4	136.7 ± 20.5	136.9 ± 13.9
**Total energy (kJ/day)**					
Mean ± SD	8461.8 ± 2033.6	8359.6 ± 2108.7	8854.4 ± 1955.4	8467.3 ± 1900.7	8168.8 ± 2184.7
**Total fat (g/day)**					
Mean ± SD	76.1 ± 23.1	74.2 ± 23.8	83.4 ± 22.6	75.5 ± 21.0	71.4 ± 24.2
**Red meat (g/day)**					
Mean ± SD	39.8 ± 32.3	34.0 ± 35.1	44.9 ± 36.6	44.7 ± 29.0	35.8 ± 28.0
**Dairy (g/day)**					
Mean ± SD	288.0 ± 163.5	277.3 ± 149.1	298.7 ± 192.4	290.9 ± 170.0	285.2 ± 144.8
**Smoking status**					
Current	9 (6)	3 (8)	2 (6)	2 (6)	2 (6)
Former (%)	76 (54)	16 (44)	19 (54)	23 (64)	18 (51)
Never (%)	57 (40)	17 (47)	14 (40)	11 (31)	15 (43)
**Physical activity**					
Inactive (%)	58 (41)	13 (36)	17 (49)	15 (42)	13 (37)
Moderately inactive (%)	36 (25)	8 (22)	9 (26)	6 (17)	13 (37)
Moderately active (%)	18 (13)	4 (11)	2 (6)	8 (22)	4 (11)
Active (%)	30 (21)	11 (31)	7 (20)	7 (19)	5 (14)
**Social class**					
Professional (1) (%)	13 (10)	5 (14)	3 (9)	3 (9)	2 (6)
Technical (2) (%)	55 (40)	14 (40)	12 (36)	17 (50)	12 (35)
Clerical NM (3.1) (%)	23 (17)	6 (17)	6 (18)	4 (12)	7 (21)
Clerical M (3.2) (%)	26 (19)	7 (20)	6 (18)	4 (12)	9 (26)
Semi-skilled (4) (%)	16 (12)	3 (9)	4 (12)	5 (15)	4 (12)
Unskilled (5) (%)	3 (2)	0 (0)	2 (6)	1 (3)	0 (0)
**Aspirin use over 3 months**					
No (%)	127 (89)	30 (83)	34 (97)	31 (86)	32 (91)
Yes (%)	15 (11)	6 (17)	1 (3)	5 (14)	3 (9)
**Family history of cancer**					
Yes (%)	67 (47)	18 (50)	17 (49)	16 (44)	16 (46)
No (%)	75 (53)	18 (50)	18 (51)	20 (56)	19 (54)
**Education level**					
High (%)	86 (61)	21 (58)	22 (63)	24 (67)	19 (54)
Low (%)	56 (39)	15 (42)	13 (37)	12 (33)	16 (46)

In this analysis mean total RFU levels were much higher among CRC cases (9534 ± 24561) compared to controls (4157 ± 6829). In multivariable analyses of these summed values, we observed a positive association between total anti-Neu5Gc IgG antibody levels and colorectal cancer. Individuals in the top quartile of anti-Neu5Gc IgG antibody concentrations had nearly three times the risk of colorectal cancer compared to those in the bottom quartile (OR: 2.98, 95% CI: 0.80, 11.09) (Table 6). Given the suggestive trend across quartiles and the skewed distribution of glycans, we also modeled it as a continuous variable: the OR (95% CI) for colorectal cancer per unit increase in log-transformed standardized sum of glycans is 1.46 (1.07, 1.99) (p-value = 0.02). Adding quartiles of alcohol as an additional covariate to the model did not make any substantial difference to the result.

**Table 6:** Matched logistic regression^a^ models for the sum of antibodies against all Neu5Gc-glycans measured in quartiles, and log transformed sum of antibodies against all Neu5Gc-glycans in the EPIC-Norfolk cohort.

	Colorectal cancer OR (95% CI)	p value
**Model 1, unadjusted**		
Quintile 1 (ref)	1	-
Quartile 2	1.23 (0.45–3.40)	0.685
Quartile 3	1.42 (0.53–3.78)	0.485
Quartile 4	1.51 (0.57–4.00)	0.411
Trend over quartiles	1.15 (0.85–1.54)	0.371
**Model 2, adjusted for study covariates**^**b**^		
Quintile 1 (ref)	1	-
Quartile 2	1.54 (0.44–5.35)	0.500
Quartile 3	1.74 (0.51–6.01)	0.379
Quartile 4	2.98 (0.80–11.09)	0.104
Trend over quartiles	1.40 (0.93–2.09)	0.105
**Model 3, log sum of antibodies against all Neu5Gc-glycans, unadjusted**		
	1.28 (1.01–1.61)	0.040
**Model 4, log sum of antibodies against all Neu5Gc-glycans, adjusted for study covariates**^b^		
	1.46 (1.07–1.99)	0.016

^a^ Cases matched to controls on date of birth, date of health examination and sex

^b^ Adjusted for body mass index, height, smoking status, physical activity, aspirin use over three months, education level, social class and family history of cancer

**Fig 6:**
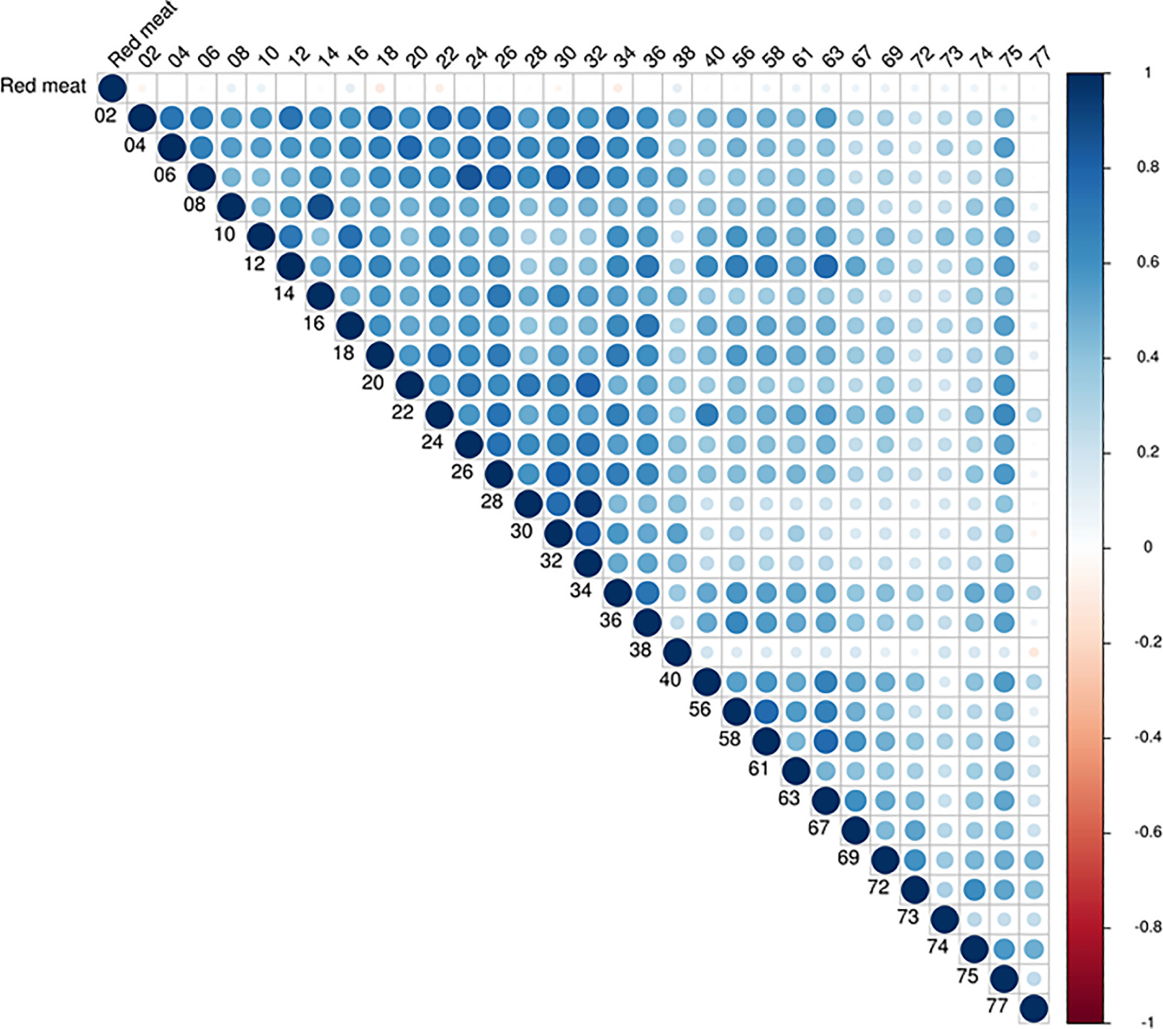
No correlation in heatmap matrix of antibodies against 31 individual Neu5Gc-glycans and red meat consumption in the EPIC-Norfolk samples studied for CRC Risk. The top and left side of the figure represents the individual glycans on the microarray, with each number representing the glycan ID. Each colored dot represents the correlation between the anti-Neu5Gc IgG against the individual glycans and red meat consumption. Black represents a correlation of 1, shades of blue are between 0 and 1, while shades of red are correlations between 0 and -1.

Notably, as with the ELISA assays, there was no apparent association between red meat intake and the sum of glycans (Table 7) or individual glycans (Fig 6) among either cases or controls in the EPIC-Norfolk study. Fig 6 shows the Spearman’s correlation heatmap of the 31 individual glycans and red meat for cases and controls combined. It attempts to summarize the correlation matrix in a compact visual format. Red meat correlations with each glycan (the top row) are shown as almost white meaning the correlations are close to zero. The remaining rows show the correlation of each glycan with every other. Almost all glycan correlations are positive, appearing as light or dark blue on the diagram. Glycans numbered 2 to 36 are strongly correlated with each other while those numbered 38 to 77 generally show weaker correlations. Glycan 77 is weakly correlated to most others.

**Table 7:** Linear regression models for the log transformed sum of antibodies against all Neu5Gc-glycans against food diary red meat for cases and controls separately in the EPIC-Norfolk cohort.

	Beta (95% CI)	p-value
**Age and sex adjusted**		
Model 1: Controls	0.009 (-0.004–0.021)	0.185
Model 2: Cases	-0.006 (-0.017–0.005)	0.299
**Multivariate adjusted**^a^		
Model 3: Controls	0.008 (-0.007–0.022)	0.286
Model 4: Cases	-0.002 (-0.016–0.011)	0.713

^a^Adjusted for body mass index, height, smoking status, physical activity, aspirin use over three months, education level, social class and family history of cancer.

## Discussion

Taken together, our data indicate that while ELISA measurement of IgG antibodies directed against limited sets of Neu5Gc-glycans show a markedly discontinuous skewed distribution in populations, they do not provide clear associations with red-meat related diseases. There was no association between anti-Neu5Gc antibodies and CAD risk when measured by such assays. In contrast to these important negative results, the sum of all antibody titers against more than 30 Neu5Gc-glycans on a glycan array was significantly associated with higher colorectal cancer risk.

The chronic inflammation induced by antibodies is generally related to complement deposition and to attraction of Fc-receptor-positive innate immune cells [78–81]. Given that epithelial and endothelial cells (where diet-derived Neu5Gc tends to accumulate) are expected to display a wide variety of sialic acid-bearing epitopes, it is actually not surprising that it is the sum total of all antibodies that showed a positive association with disease risk—rather than antibodies directed against a specific glycan. Although the sample size for this colorectal cancer case-control study was small and statistical power was limited, the results warrant a thorough investigation between the total anti-Neu5Gc immune response and colorectal cancer risk. Replication in future studies will be necessary.

Notably, a strong correlation was observed between anti-Neu5Gc IgG titers in the ELISA assay and the fraction inhibitable by Neu5Gcα2Me. This indicates that use of the expensive inhibitor to determine specificity is unnecessary. However, while anti-Neu5Gc IgG can be measured and followed with an ELISA screening assay, the sialoglycan microarray approach will likely be needed to accurately identify sub-populations at risk and/or unique combinations of epitopes that are associated with specific diseases. Of course, the sialoglycan microarray approach is much more expensive and time-consuming. In this regard, it is notable that the majority of individuals in the populations studied had rather low levels of antibodies detected in all of the assays. Since the distribution of levels is so highly skewed in the population, there is a possibility that only those with high levels are at risk for red meat related disease. Thus, it may be worthwhile to see if a screening ELISA assay (such as the ones described here) can be used to focus attention on those individuals whose total antibody levels are in the upper quartile or quintile of the population distribution. However, the more expensive and time-consuming slide array studies will likely still be required to obtain more precision.

Consistent with our earlier data regarding early childhood emergence of anti-Neu5Gc antibodies [21], we did not find a correlation with dietary red meat intake in 338 adult women. We queried usual adult red meat consumption; given that levels of anti-Neu5Gc antibodies are determined earlier in life, i.e., upon first exposure to red meat and/or represent cumulative exposure over a lifetime, this result is not unexpected. We also lacked information regarding introduction of solid foods in infancy for adult participants in these studies, which could have provided further insights into the discontinuous distribution of antibodies we observed.

Despite a strong rationale for associations with disease risk [20,21,68,68,75,82] and recent suggestive reports from us and others studying various diseases and clinical situations [67,83–90], such simple ELISA assays directed at specific Neu5Gc-containing epitopes, or even the limited mixtures of epitopes studied here did not give definitive correlations with disease risk. However, in retrospect this result is logical when considering the disease mechanism involved. In any given cancer or inflammatory cardiovascular lesion, a multitude of varied epitopes presenting the non-human Neu5Gc sialic acid are expected. Given that each individual has a distinct profile of antibodies it is consistent that the total level of all antibodies combined has the closest correlation with inflammation and disease risk. Our finding of a positive association of total anti-Neu5Gc antibodies with CRC risk warrants confirmation in larger prospective studies.

Further work harnessing the utility of these anti-Neu5Gc antibodies as biomarkers in red meat-associated diseases must consider such diversity in individual antibody profiles against different Neu5Gc-bearing glycans. Traditional ELISA assays directed against Neu5Gc alone, or against specific Neu5Gc-glycans may not be adequate to define risk associations. Further research on the association of anti-Neu5Gc IgG and red meat-related pathology will have to utilize either the complex and expensive slide array method, or perhaps a novel approach to generating a mixed target containing most of the epitopes.

## Supporting information

S1 Table List of Neu5Ac and Neu5Gc terminated glycans, ID and structure used in the microarray. (DOCX)

S2 Table Mean and median reactivity for individual Neu5Gc-glycans in colorectal cancer cases from the EPIC-Norfolk cohort. (DOCX)

S3 Table Descriptive characteristics for cases of colorectal cancer and matched controls from the EPIC-Norfolk cohort. (DOCX)

S4 Table Correlation coefficients of each analyte with coronary artery disease variables. (DOCX)

S1 Dataset Raw data for all experiments (ELISA and microarray). (XLSX)
