# Supplementary material for: Polyclonal human antibodies against glycans bearing red meat-derived non-human sialic acid N-glycolylneuraminic acid are stable, reproducible, complex and vary between individuals: Total antibody levels are associated with colorectal cancer risk
Source: PLoS One. 2018 Jun 18;13(6):e0197464. doi: 10.1371/journal.pone.0197464 (PMC6005533; doi:10.1371/journal.pone.0197464)
Supplement: S1 Table — (DOCX) [file pone.0197464.s001.docx]

**Supplemental Table 1: Neu5Ac and Neu5Gc terminated glycans: ID and structure used in microarray**

| **ID** | **Glycan structure** |
| --- | --- |
| 01 | Neu5,9Ac_2_α3Galβ4GlcNAcβR1 |
| 02 | **Neu5Gc**9Acα3Galβ4GlcNAcβR1 |
| 03 | Neu5,9Ac_2_α6Galβ4GlcNAcβR1 |
| 04 | **Neu5Gc**9Acα6Galβ4GlcNAcβR1 |
| 05 | Neu5Acα6GalNAcαR1 |
| 06 | **Neu5Gc**α6GalNAcαR1 |
| 07 | Neu5,9Ac_2_α3Galβ3GlcNAcβR1 |
| 08 | **Neu5Gc**9Acα3Galβ3GlcNAcβR1 |
| 09 | Neu5,9Ac_2_α3Galβ3GalNAcαR1 |
| 10 | **Neu5Gc**9Acα3Galβ3GalNAcαR1 |
| 11 | Neu5Acα3Galβ4GlcNAcβR1 |
| 12 | **Neu5Gc**α3Galβ4GlcNAcβR1 |
| 13 | Neu5Acα3Galβ3GlcNAcβR1 |
| 14 | **Neu5Gc**α3Galβ3GlcNAcβR1 |
| 15 | Neu5Acα3Galβ3GalNAcαR1 |
| 16 | **Neu5Gc**α3Galβ3GalNAcαR1 |
| 17 | Neu5Acα6Galβ4GlcNAcβR1 |
| 18 | **Neu5Gc**α6Galβ4GlcNAcβR1 |
| 19 | Neu5Acα6Galβ4GlcβR1 |
| 20 | **Neu5Gc**α6Galβ4GlcβR1 |
| 21 | Neu5Acα3Galβ4GlcβR1 |
| 22 | **Neu5Gc**α3Galβ4GlcβR1 |
| 23 | Neu5,9Ac_2_α6GalNAcαR1 |
| 24 | **Neu5Gc**9Acα6GalNAcαR1 |
| 25 | Neu5Acα3GalβR1 |
| 26 | **Neu5Gc**α3GalβR1 |
| 27 | Neu5Acα6GalβR1 |
| 28 | **Neu5Gc**α6GalβR1 |
| 29 | Neu5,9Ac_2_α3GalβR1 |
| 30 | **Neu5Gc**9Acα3GalβR1 |
| 31 | Neu5,9Ac_2_α6GalβR1 |
| 32 | **Neu5Gc**9Acα6GalβR1 |
| 33 | Neu5Acα3Galβ3GalNAcβR1 |
| 34 | **Neu5Gc**α3Galβ3GalNAcβR1 |
| 35 | Neu5,9Ac_2_α3Galβ3GalNAcβR1 |
| 36 | **Neu5G**c9Acα3Galβ3GalNAcβR1 |
| 37 | Neu5,9Ac_2_α6Galβ4GlcβR1 |
| 38 | **Neu5Gc**9Ac6Galβ4GlcβR1 |
| 39 | Neu5,9Ac_2_α3Galβ4GlcβR1 |
| 40 | **Neu5Gc**9Ac3Galβ4GlcβR1 |
| 41 | Neu5Acα8Neu5Acα3Galβ4GlcβR1 |
| 42 | Neu5Acα8Neu5Acα8Neu5Acα3Galβ4GlcβR1 |
| 43 | Galβ4GlcβR1 |
| 45 | Galβ4GlcNAcβR1 |
| 47 | GalNAcαR1 |
| 51 | Galβ3GalNAcβR1 |
| 52 | Galβ3GalNAcαR1 |
| 53 | Galβ3GlcNAcβR1 |
| 54 | Galβ4GlcNAc6SβR1 |
| 55 | Neu5Acα3Galβ4(Fucα3)GlcNAcβR1 |
| 56 | **Neu5Gc**α3Galβ4(Fucα3)GlcNAcβR1 |
| 57 | Neu5Acα3Galβ4(Fucα3)GlcNAc6SβR1 |
| 58 | **Neu5Gc**α3Galβ4(Fucα3)GlcNAc6SβR1 |
| 60 | Neu5Acα3Galβ3GlcNAcβ3Galβ4GlcβR1 |
| 61 | **Neu5Gc**α3Galβ3GlcNAcβ3Galβ4GlcβR1 |
| 62 | Neu5Acα3Galβ4GlcNAc6SβR1 |
| 63 | **Neu5Gc**α3Galβ4GlcNAc6SβR1 |
| 64 | Neu5Acα8Neu5Acα3Galβ4GlcβR2 |
| 65 | Neu5Acα8Neu5Acα8Neu5Acα3Galβ4GlcβR2 |
| 66 | Neu5Acα6(Neu5Acα3)Galβ4GlcβR1 |
| 67 | Neu5Acα6(**Neu5Gc**α3)Galβ4GlcβR1 |
| 68 | Neu5Acα6(Kdnα3)Galβ4GlcβR1 |
| 69 | **Neu5Gc**α8Neu5Acα3Galβ4GlcβR1 |
| 70 | Kdnα8Neu5Acα3Galβ4GlcβR1 |
| 72 | Neu5Acα8Neu5Gcα3Galβ4GlcβR1 |
| 73 | Neu5Acα8Neu5Gcα6Galβ4GlcβR1 |
| 74 | Kdnα8Neu5Gcα3Galβ4GlcβR1 |
| 75 | **Neu5Gc**α8Neu5Gcα3Galβ4GlcβR1 |
| 76 | Neu5Acα8Neu5Acα6Galβ4GlcβR1 |
| R1 = O(CH_2_)_3_NH_2_ | |
| R2 = O(CH_2_)_3_NHCOCH_2_(OCH_2_CH_2_)_6_NH_2_ | |
