# Supplementary material for: Polyclonal human antibodies against glycans bearing red meat-derived non-human sialic acid N-glycolylneuraminic acid are stable, reproducible, complex and vary between individuals: Total antibody levels are associated with colorectal cancer risk
Source: PLoS One. 2018 Jun 18;13(6):e0197464. doi: 10.1371/journal.pone.0197464 (PMC6005533; doi:10.1371/journal.pone.0197464)
Supplement: S2 Table — (DOCX) [file pone.0197464.s002.docx]

**Supplemental Table 2. Mean and median reactivity for individual Neu5Gc-glycans in colorectal cancer cases from the EPIC-Norfolk cohort.**

| Glycan Number | Case Mean ± SD | Case Median (IRQ) | Control Mean ± SD | Control Median (IRQ) |
| --- | --- | --- | --- | --- |
| **Glycan 02** | 293.6 ± 946.4 | 10.2 (0.0 - 100.8) | 78.0 ± 166.4 | 0.0 (0.0 - 58.5) |
| **Glycan 04** | 165.5 ± 519.6 | 5.0 (0.0 - 104.9) | 71.7 ± 182.7 | 0.0 (0.0 - 19.9) |
| **Glycan 06** | 260.3 ± 475.7 | 85.5 (24.9 - 254.0) | 153.8 ± 201.8 | 96.0 (13.9 - 208.1) |
| **Glycan 08** | 465.3 ± 1263.9 | 52.0 (0.0 - 188.2) | 110.5 ± 216.3 | 0.0 (0.0 - 107.2) |
| **Glycan 10** | 325.9 ± 1210.5 | 0.0 (0.0 - 36.0) | 79.2 ± 178.2 | 0.0 (0.0 - 41.4) |
| **Glycan 12** | 412.9 ± 1371.2 | 0.0 (0.0 - 51.0) | 69.8 ± 203.1 | 0.0 (0.0 - 0.0) |
| **Glycan 14** | 353.6 ± 850.2 | 67.0 (29.1 - 207.9) | 138.0 ± 194.4 | 56.5 (0.0 - 173.8) |
| **Glycan 16** | 298.6 ± 1134.5 | 0.0 (0.0 - 47.0) | 51.6 ± 127.8 | 0.0 (0.0 - 10.6) |
| **Glycan 18** | 245.8 ± 881.3 | 0.0 (0.0 - 31.4) | 90.1 ± 246.1 | 0.0 (0.0 - 19.1) |
| **Glycan 20** | 121.9 ± 208.0 | 34.2 (0.0 - 144.9) | 105.2 ± 233.5 | 8.5 (0.0 - 88.4) |
| **Glycan 22** | 456.9 ±1367.9 | 48.0 (0.0 - 206.9) | 301.1 ± 1173.6 | 16.0 (0.0 - 173.9) |
| **Glycan 24** | 304.2 ± 817.3 | 44.2 (0.2 - 176.9) | 115.6 ± 209.4 | 24.2 (0.0 - 121.1) |
| **Glycan 26** | 328.6 ± 962.4 | 57.0 (13.9 - 152.2) | 121.3 ± 227.5 | 17.5 (0.0 - 113.8) |
| **Glycan 28** | 227.3 ± 246.7 | 172.2 (38.2 - 322.5) | 204.5 ± 261.3 | 98.8 (25.1 - 281.1) |
| **Glycan 30** | 241.5 ± 424.2 | 104.8 (35.1 - 235.8) | 193.3 ± 469.2 | 83.5 (28.5 - 183.4) |
| **Glycan 32** | 278.4 ± 401.5 | 139.8 (48.1 - 326.2) | 218.3 ± 417.4 | 87.8 (13.4 - 236.9) |
| **Glycan 34** | 331.0 ± 1192.8 | 10.5 (0.0 - 53.1) | 96.2 ± 216.1 | 0.0 (0.0 - 47.6) |
| **Glycan 36** | 373.3 ± 1474.1 | 0.0 (0.0 - 26.1) | 85.3 ± 272.1 | 0.0 (0.0 - 6.9) |
| **Glycan 38** | 563.6 ± 703.6 | 272.5 (105.1 - 687.6) | 488.4 ± 679.8 | 303.0 (74.8 - 629.0) |
| **Glycan 40** | 534.7 ± 1664.0 | 13.5 (0.0 - 133.2) | 233.8 ± 678.9 | 0.0 (0.0 - 140.4) |
| **Glycan 56a** | 373.8 ± 1571.4 | 0.0 (0.0 - 0.6) | 86.0 ± 313.7 | 0.0 (0.0 - 3.9) |
| **Glycan 58** | 302.2 ± 999.9 | 0.0 (0.0 - 10.5) | 91.4 ± 382.6 | 0.0 (0.0 - 0.1) |
| **Glycan 61** | 395.2 ± 1455.3 | 0.0 (0.0 - 91.0) | 162.6 ± 732.4 | 0.0 (0.0 - 13.5) |
| **Glycan 63** | 643.1 ±1940.6 | 0.0 (0.0 - 130.1) | 148.0 ± 408.4 | 0.0 (0.0 - 33.4) |
| **Glycan 67** | 192.1 ±723.4 | 0.0 (0.0 - 8.2) | 64.2 ± 318.1 | 0.0 (0.0 - 0.0) |
| **Glycan 69** | 218.2 ±798.0 | 0.0 (0.0 - 16.0) | 86.5 ± 265.9 | 0.0 (0.0 - 7.5) |
| **Glycan 72** | 110.8 ± 401.8 | 0.0 (0.0 - 0.0) | 80.4 ± 349.7 | 0.0 (0.0 - 0.0) |
| **Glycan 73** | 73.3 ± 387.0 | 0.0 (0.0 - 0.0) | 53.0 ± 313.3 | 0.0 (0.0 - 0.0) |
| **Glycan 74** | 137.1 ± 364.6 | 8.3 (0.0 - 56.4) | 105.2 ± 383.6 | 0.0 (0.0 - 17.9) |
| **Glycan 75** | 306.8 ± 902.5 | 40.5 (0.0 - 133.9) | 148.1 ± 338.7 | 10.0 (0.0 - 97.2) |
| **Glycan 77** | 198.1 ± 676.6 | 0.0 (0.0 - 50.2) | 125.9 ± 385.7 | 0.0 (0.0 - 48.1) |
|  | **Sum of antibodies against all Neu5Gc-glycans** | | | |
|  | 9533.6  ± 24561.4 | 2209.0  (1302.8 - 4973.5) | 4156.9  ± 6828.6 | 1805.2  (797.2 - 3407.0) |
