# Supplementary material for: Polyclonal human antibodies against glycans bearing red meat-derived non-human sialic acid N-glycolylneuraminic acid are stable, reproducible, complex and vary between individuals: Total antibody levels are associated with colorectal cancer risk
Source: PLoS One. 2018 Jun 18;13(6):e0197464. doi: 10.1371/journal.pone.0197464 (PMC6005533; doi:10.1371/journal.pone.0197464)
Supplement: S3 Table — (DOCX) [file pone.0197464.s003.docx]

**Supplemental Table 3. Descriptive characteristics for cases of colorectal cancer and matched controls from the EPIC-Norfolk cohort**

|  | Case  (n=71; 50.0%) | Control  (n=71; 50.0%) |
| --- | --- | --- |
| **Age, years** | | |
| Mean ± SD | 67.0 ± 6.1 | 67.1 ± 6.1 |
| **Sex** | | |
| Men (%) | 49 (50) | 49 (50) |
| Women (%) | 22 (50) | 22 (50) |
| **Body mass index, kg/m²** | | |
| Mean ± SD | 26.7 ± 3.2 | 26.6 ± 3.5 |
| **Weight, kg** | | |
| Mean ± SD | 76.3 ± 12.8 | 76.8 ± 11.8 |
| **Height, cm** | | |
| Mean ± SD | 168.8 ± 8.8 | 169.8 ± 9.1 |
| **Units of Alcohol per week** | | |
| Mean ± SD | 8.7 ± 9.4 | 6.9 ± 7.4 |
| **Total cholesterol** | | |
| Mean ± SD | 6.2 ± 1.3 | 6.6 ± 1.2 |
| **Systolic blood pressure** | | |
| Mean ± SD | 136.8 ± 17.8 | 138.9 ± 18.6 |
| **Total energy (kJ/day)** | | |
| Mean ± SD | 8304.3 ± 2036.8 | 8619.4 ± 2032.7 |
| **Total fat (g/day)** | | |
| Mean ± SD | 72.7 ± 20.5 | 79.4 ± 25.1 |
| **Red meat (g/day)** | | |
| Mean ±SD | 40.0 ± 33.0 | 39.5 ± 31.9 |
| **Dairy (g/day)** | | |
| Mean ±SD | 260.6 ± 158.6 | 315.3 ± 164.9 |
| **Smoking status** | | |
| Current (%) | 2 (22) | 7 (78) |
| Former (%) | 41 (54) | 35 (46) |
| Never (%) | 28 (49) | 29 (51) |
| **Physical activity** | | |
| Inactive (%) | 28 (48) | 30 (52) |
| Moderately inactive (%) | 17 (47) | 19 (53) |
| Moderately active (%) | 12 (67) | 6 (33) |
| Active (%) | 14 (47) | 16 (53) |
| **Social class** | | |
| Professional (1) (%) | 6 (46) | 7 (54) |
| Technical (2) (%) | 27 (49) | 28 (51) |
| Clerical NM (3.1) (%) | 16 (70) | 7 (30) |
| Clerical M (3.2) (%) | 8 (31) | 18 (69) |
| Semi-skilled (4) (%) | 8 (50) | 8 (50) |
| Unskilled (5) (%) | 1 (33) | 2 (67) |
| **Aspirin use over 3 months** | | |
| No (%) | 62 (49) | 65 (51) |
| Yes (%) | 9 (60) | 6 (40) |
| **Family history of cancer** | | |
| Yes (%) | 31 (46) | 36 (54) |
| No (%) | 40 (53) | 35 (47) |
| **Education level** | | |
| High (%) | 42 (49) | 44 (51) |
| Low | 29 (52) | 27 (48 |
| **Anti-Neu5GcIgG** |  |  |
| Quartile 1 | 16 (44.4) | 20 (55.6) |
| Quartile 2 | 17 (48.6) | 18 (51.4) |
| Quartile 3 | 19 (52.8) | 17 (47.2) |
| Quartile 4 | 19 (54.3) | 16 (45.7) |
