# Supplementary material for: Polyclonal human antibodies against glycans bearing red meat-derived non-human sialic acid N-glycolylneuraminic acid are stable, reproducible, complex and vary between individuals: Total antibody levels are associated with colorectal cancer risk
Source: PLoS One. 2018 Jun 18;13(6):e0197464. doi: 10.1371/journal.pone.0197464 (PMC6005533; doi:10.1371/journal.pone.0197464)
Supplement: S4 Table — (DOCX) [file pone.0197464.s004.docx]

**Supplemental Table 4. Correlation coefficients of each analyte with coronary artery disease variables**

|  | Anti-Neu5Gc IgG against Neu5Gc-alpha-PAA (µg/mL) | Anti-Neu5Gc IgG against Neu5Gc2-6Lac-HAS (µg/mL) | Total anti-Neu5Gc IgG against mouse serum Neu5Gc-terminated glycans (µg/mL)† | Neu5Gc Inhibitable IgG against mouse serum Neu5Gc-terminated glycans (µg/mL)† |
| --- | --- | --- | --- | --- |
| Age | 0.02 | 0.02 | 0.09 | 0 |
| BMI | 0.06 | -0.02 | 0.02 | -0.01 |
| Systolic BP | 0.05 | -0.03 | -0.01 | -0.04 |
| Cholesterol | -0.04 | -0.05 | -0.04 | -0.05 |
| WBC | 0.05 | -0.08 | 0.02 | 0 |
| CRP | 0.08 | 0 | -0.01 | -0.02 |
